# Glucose‐lowering effect of *Gryllus bimaculatus* powder on streptozotocin‐induced diabetes through the AKT/mTOR pathway

**DOI:** 10.1002/fsn3.1323

**Published:** 2019-12-11

**Authors:** Seon‐Ah Park, Geum‐Hwa Lee, Hwa‐Young Lee, The‐Hiep Hoang, Han‐Jung Chae

**Affiliations:** ^1^ Non‐Clinical Evaluation Center Biomedical Research Institute Chonbuk National University Hospital Jeonju Chonbuk South Korea; ^2^ Department of Pharmacology and Institute of New Drug Development School of Medicine Chonbuk National University Jeonju Chonbuk South Korea

**Keywords:** AKT/mTOR, Bax, Bcl2, Diabetes, *Gryllus bimaculatus*

## Abstract

This study was carried out to elucidate the antidiabetic effects of *Gryllus bimaculatus* powder using a streptozotocin (STZ)‐induced rat model of type I diabetes. Administration of the insect powder significantly rescued representative diabetes markers (i.e., insulin and C‐peptide) in STZ‐treated rats. Improved glucose tolerance test (GTT) and insulin tolerance test (ITT) results were also observed, indicating that *Gryllus bimaculatus* powder exerts antidiabetic effects. *Gryllus bimaculatus* powder administration rescued STZ‐induced alterations in both islet morphology and insulin staining patterns. The extract increased antiapoptotic Bcl2 expression and decreased proapoptotic Bax and active caspase 3 expressions. In addition, the *Gryllus bimaculatus* powder supplementation enhanced AKT/mTOR pathway, a key marker of the state of anabolic metabolism, and its downstream effector, mTOR. Collectively, our results suggest that *Gryllus bimaculatus* contributes to the maintenance of pancreatic β‐cell function and morphology against a diabetic state through the regulations against apoptosis and anabolic metabolism.

## Graphical abstract

This study suggests the antidiabetic effects of *Gryllus bimaculatus* powder in a streptozotocin‐induced rat model. *Gryllus bimaculatus* powder improves diabetic conditions via the AKT/mTOR pathway pathway in a streptozotocin‐induced diabetic animal model. This study suggests that *G. bimaculatus* powder contributes to the maintenance of pancreatic β‐cell function and morphology in a streptozotocin‐induced diabetic animal model.

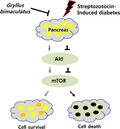

## 1 INTRODUCTION

Diabetes mellitus refers to a group of metabolic disorders that include diseases exhibiting increased blood sugar levels resulting from inadequate insulin secretion or insulin resistance (Novikova et al., 2013; van Belle, Coppieters, & Herrath, 2011). Type 1 diabetes (T1D) is a chronic autoimmune disease characterized by selective autoimmune‐mediated destruction of β‐cells in pancreatic islets, gradually leading to absolute insulin deficiency (Novikova et al., 2013; van Belle et al., 2011). Life‐long insulin administration is necessary for patients with T1D. To help manage diabetes and improve the quality of life and nutritional balance of T1D patients, nutrition‐based functional foods are recommended.

A streptozotocin (STZ)‐induced animal model has been suggested as an appropriate method to examine the efficacy of foods that can ameliorate T1D (Deeds et al., 2011; Shen et al., 2012; Zhang et al., 2014). STZ is a glucosamine–nitrosourea compound that enters pancreatic β‐cells through oxidation, leading to the formation of superoxide radicals; as a result, hydrogen peroxide and hydroxyl radicals are produced (Eleazu, Eleazu, Chukwuma, & Essien, 2013; Lenzen, 2008), and STZ inhibits aconitase activity and causes the release of toxic nitrogen oxides that damage DNA. Most importantly, STZ toxicity results in pancreatic β‐cell necrosis (Lenzen, 2008; Sakuraba et al., 2002).

Among the highly nutritious functional food sources, insects such as crickets are ranked 4th globally. Cricket production efficiency is relatively high (80%) compared to beef (40%), pork (55%), and poultry (55%). Furthermore, insects are emerging as an alternative to animal protein (Kouřimská & Adámková, 2016). Ahn et al. (2005) reported that the cricket *Gryllus bimaculatus* contains unsaturated fatty acids that can be used both as food and as a remedy for fever, diarrhea, kidney stones, and hypertension. In addition, reports have suggested that the ethanol extract of *Gryllus bimaculatus* is not toxic to humans (Lee et al., 2016; Ryu et al., 2016). Consequently, this study was conducted to determine whether intake of *Gryllus bimaculatus* powder could contribute to the recovery of pancreatic cell function and its associated antidiabetic condition in an STZ‐induced rat model of T1D.

## 2 MATERIALS AND METHODS

### 2.1 Materials

STZ, glucose, insulin, and hematoxylin and eosin Y solution were purchased from Sigma‐Aldrich (St. Louis, MO, USA). A C‐peptide ELISA Kit was purchased from BioVision (Eugene, OR, USA). A rat/mouse insulin ELISA Kit was purchased from Merck Millipore (EMD Millipore, Darmstadt, Germany). For immunoblotting, antibodies against β‐actin, p‐AKT, and Bax were purchased from Santa Cruz Biotechnology (Santa Cruz, CA, USA). AKT, p‐AKT, p‐p70S6K, 4EBP1, p‐4EBP1, mTOR, p‐mTOR, Bcl2, insulin, and cleaved caspase 3 were purchased from Cell Signaling Technology (Beverly, MA, USA). A commercial brand of *Gryllus bimaculatus* powder under the name “D&D (Diabetes & Dietary, Inventor: Dr Lee Sam Goo, South Korea)” was obtained from 239bio Inc. (Ixsan, Chonbuk, South Korea). To create the powder, the growth period of *Gryllus bimaculatus* was limited to a maximum of 35 days. Crickets were subjected to a 3‐day defecation period, washed three times in distilled water, and then freeze‐dried. The freeze‐dried *Gryllus bimaculatus* were homogenized, and the powder was stored at −20°C for 4 weeks. Powder manufacturing is based on patents belonging to 239bio Inc., Korea, with the following registration numbers: 10–1686179, 10–1663202, 10–1702851, 10–1716766, 10–1716763, 10–1773851, and 10–1809451).

### 2.2 Animals

Eight‐week‐old male *SD* rats were purchased from Saeron Bio Inc. (Uiwang‐si, Gyeonggi‐do, Korea). All animals were housed at 18–25°C under a 12‐hr light/dark cycle and allowed ad libitum access to food and water. After 1 week of acclimatization, the rats were injected intraperitoneally (IP) with a single dose of freshly prepared STZ (65 mg/kg, Sigma‐Aldrich; 0.05 M citrate buffer; pH 4.5) to induce T1D. The control group was injected with an equal volume containing only citrate buffer. Diabetes was confirmed 7 days postinjection by measuring blood glucose levels with an Accu‐Chek glucometer (Roche, Boston, MA, USA). Blood glucose levels were measured once a week on the day prior to *Gryllus bimaculatus* powder administration (Ryu et al., 2016). The control group was fed only the highest dose (6.5 g/kg) of *Gryllus bimaculatus* powder, while the diabetic group was fed various doses (1.63, 3.25, and 6.5 g/kg) twice a day (10:30 and 16:00). The control group was fed an equal volume of water.

### 2.3 Blood glucose measurements, intraperitoneal glucose tolerance tests, and insulin tolerance tests

Blood glucose measurements, intraperitoneal glucose tolerance tests (GTTs), and insulin tolerance tests (ITT) were performed as previously described (Cho, Zhou, Sheng, & Rui, 2011). Briefly, blood glucose levels were measured weekly in rat tail blood samples, starting with oral administration of *Gryllus bimaculatus* powder. GTTs were performed 2 days before euthanasia, and the assays were performed at 09:00 after 12 hr of fasting (food was withdrawn at 21:00 the previous night). A 10% glucose solution (1 g/kg) was given as a bolus IP injection, and tail blood samples were collected at 0, 15, 30, 45, 60, 90, and 120 min postadministration for determination of blood glucose levels. A similar procedure was performed for the ITTs at 08:00 after 4 hr of fasting (food was withdrawn at 04:00). Insulin (0.75 U/kg) was administered as a bolus IP injection, and blood glucose levels were determined at 0, 15, 30, 45, 60, 90, and 120 min postinjection.

### 2.4 Immunohistochemical staining

Immunohistochemical staining was performed as previously described (Chau et al., 2017; Franko et al., 2016; Song et al., 2010). For the analysis, rat pancreases were fixed in a 3.7% formaldehyde solution, dehydrated in a graded ethanol series, embedded in paraffin (Leica, Wetzlar, Germany), and sectioned into 4‐µm slices. For H&E staining of the pancreas, sections were first stained with hematoxylin for 2 min and then eosin (Sigma‐Aldrich) for 5 min after a 10‐min wash. For insulin staining in the pancreas islets, sections were incubated with an insulin antibody (1:100, Santa Cruz Biotechnology). Insulin expression was detected with 3‐amino‐9‐ethylcarbazole (AEC; Dako, Santa Clara, CA, USA), and the nucleus was then stained with hematoxylin.

### 2.5 Immunoblotting

Total protein was extracted from the pancreas using a lysis buffer (150 mM NaCl, 0.5 M Tris‐HCl, 2.5% deoxycholic acid, 10% NP‐40, and 10 mM EDTA; pH 7.4). Samples were separated by 10%–13% sodium dodecyl sulfate (SDS)–polyacrylamide gel electrophoresis (PAGE) and transferred to PVDF membranes (Bio‐Rad). The membranes were incubated with the specific primary antibodies (Bax, Bcl2, AKT, p‐AKT, p70S6 kinase, p‐p70S6 kinase, 4EBP1, p‐4EBP1, mTOR, p‐mTOR, cleaved caspase 3, and β‐actin, 1:1,000 to 1:2,000) overnight at 4°C, followed by incubation with a horseradish peroxidase–IgG‐conjugated secondary antibody for 1 hr at room temperature. The signals were visualized using X‐ray film (GE Healthcare, Amersham, Buckinghamshire, UK). Protein expression was analyzed using band detection software in ImageJ (NIH, MD, USA).

### 2.6 Statistical analysis

All values are expressed as means ± *SEM*. A *t* test was used for all groups. Statistical calculations, plotting, and curve fitting were performed using Origin 7.0 (OriginLab Co., MA, USA). A *P* value < 0.05 was considered significant.

## 3 RESULTS AND DISCUSSION

### 3.1 Effects of *Gryllus bimaculatus* powder on blood glucose, plasma C‐peptide, and plasma insulin levels in STZ‐induced diabetic rats

**Figure 1:**
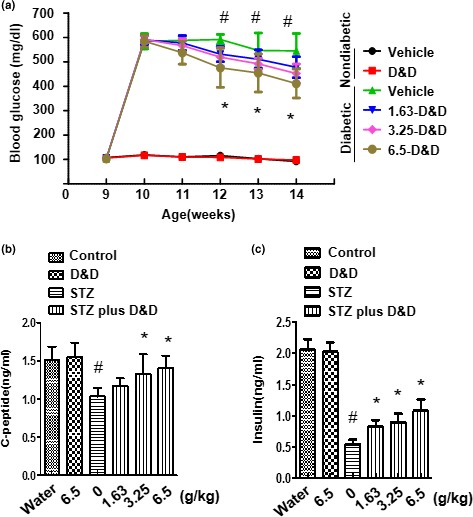
Effects of *Gryllus bimaculatus* powder on blood glucose, plasma C‐peptide, and plasma insulin levels in an STZ‐induced diabetic rat model. Eight‐week‐old rats were injected intraperitoneally with a single dose of freshly prepared STZ (65 mg/kg). The control group was injected with the same volume of citrate buffer only. After confirming the diabetic condition, the control group was fed only a high dose of the *Gryllus bimaculatus* powder (6.5 g/kg), whereas the diabetic group was fed varying doses of the powder (1.63, 3.25, and 6.5 g/kg) twice daily. The fasting glucose level was measured as described in the Materials and Methods (a). Plasma C‐peptide levels (b) and insulin levels **(**c) were measured as described in the Materials and Methods. Values are means ± *SEM*. *n* = 8; ^#^ *p* < .05 versus control group *p < .05 versus STZ groups

*Gryllus bimaculatus* powder was administered to STZ‐induced diabetic rats to confirm its effect on blood glucose. Blood glucose levels in STZ‐induced diabetic rats were significantly higher compared to the control group, whereas the levels were decreased in a concentration‐dependent manner (Figure 1a). Markers of T1D, including serum C‐peptide and insulin levels, were significantly decreased in the STZ‐induced diabetes group compared to the nondiabetic group (Figure 1b,c). The *Gryllus bimaculatus* powder‐treated group presented a dose‐dependent rescue of the levels of these markers, showing that *Gryllus bimaculatus* powder has a glucose‐lowering effect in the T1D rat model.

Administering STZ to rats resulted in increased blood glucose levels and decreased plasma or serum insulin levels (Beppu et al., 2003; Cho et al., 2011; Deeds et al., 2011; Lenzen, 2008; Szkudelski, 2001; Zhang et al., 2014). Nonclinical studies have recently been conducted to verify whether edible insects improve diabetes. Edible insects known to exert hypoglycemic effects include silkworms (*Bombyx mori*), mealworms (*Tenebrio molitor*), beetles (*Protaetia brevitarsis*), and crickets. Studies on silkworms (Rattana, Katisart, Butiman, & Sungthong, 2017) showed that fibroin and sericin in silkworm powder lowered plasma glucose levels (Rattana et al., 2017). *Gryllus bimaculatus* is reported to contain unsaturated fatty acids, essential fatty acids, and a high protein content, as well as to markedly boost immunity (Grapes, Whiting, & Dinan, 1989; Kim et al., 2016). The administration of the *Gryllus bimaculatus* extracts containing these compounds was shown to lower blood glucose levels and exert a preventive effect against the loss of β‐cells.

### 3.2 Effects of *Gryllus bimaculatus* powder on glucose and insulin tolerance states

**Figure 2:**
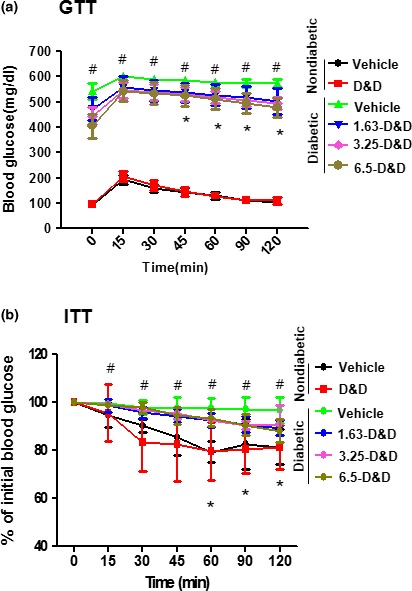
Effects of *Gryllus bimaculatus* powder on glucose (glucose tolerance test; GTT) and insulin tolerance (insulin tolerance test; ITT) states.** **Eight‐week‐old rats were injected intraperitoneally with a single dose of freshly prepared STZ (65 mg/kg). The control group was injected with an equal volume of citrate buffer only. After confirming the diabetic condition, the control group was fed a high dose of the *Gryllus bimaculatus* powder (6.5 g/kg) and the diabetic group was fed varying doses of the powder (1.63, 3.25, and 6.5 g/kg) twice daily. The GTT (a) and ITT (b) were performed as described in the Materials and Methods. Values are means ± *SEM*. *n* = 8; ^#^ *p* < .05 versus control groups; *p < .05 versus the STZ group

Impaired glucose tolerance (IGT) is a prediabetic state of hyperglycemia that is associated with insulin resistance. In the last week of the experiment, a glucose tolerance test was performed on the rats to evaluate insulin sensitivity. As shown in Figure 2a, glucose tolerance was impaired in the STZ‐induced diabetes group, but this impairment was significantly rescued in the *Gryllus bimaculatus* powder‐treated group. To determine insulin sensitivity in the presence of *Gryllus bimaculatus* powder, an insulin tolerance test was separately applied to the model. As expected, treatment with the *Gryllus bimaculatus* powder enhanced insulin sensitivity in the STZ‐induced diabetes model (Figure 2b).

Although the study was performed based on a T1D model, glucose and insulin sensitivities confirmed the response to glucose and insulin, suggesting that the *Gryllus bimaculatus* powder exerted a beneficial effect against insulin resistance (Figure 2a,b). However, only the highest dose of the *Gryllus bimaculatus* powder (6.5 g/kg) elicited a significant ameliorating effect against insulin resistance, indicating that this effect was less distinct than the other effects, such as pancreatic morphology and insulin and C‐peptide levels.

### 3.3 Effects of *Gryllus bimaculatus* powder on pancreatic function and morphology

**Figure 3:**
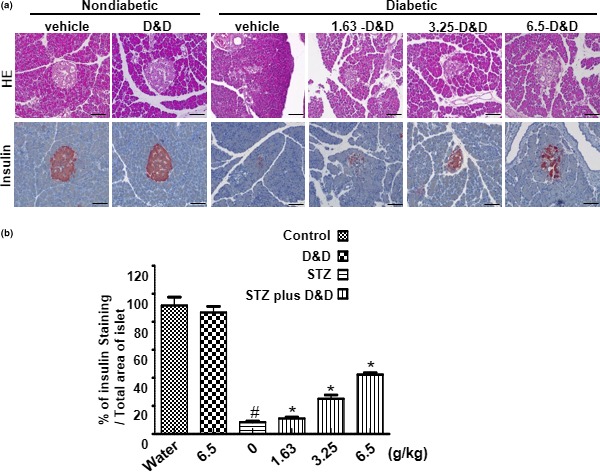
Effects of *Gryllus bimaculatus* powder on pancreatic function and morphology. Eight‐week‐old rats were injected intraperitoneally with a single dose of freshly prepared STZ (65 mg/kg). The control group was injected with an equal volume of citrate buffer only. After confirming the diabetic condition, the control group was fed a high dose of the *Gryllus bimaculatus* powder (6.5 g/kg), whereas the diabetic group was fed varying doses of the powder (1.63, 3.25, and 6.5 g/kg) twice daily. Hematoxylin and eosin (H&E) immunohistochemistry (top; ×200 magnification) and immunohistochemical staining with an anti‐insulin antibody (bottom; ×200 magnification) of isolated pancreases from the vehicle and *Gryllus bimaculatus* powder‐administered nondiabetic rats and from the vehicle and *Gryllus bimaculatus* powder‐administered diabetic rats (a). Quantification analysis of the positive insulin staining pancreatic islets (b). Values are means ± *SEM*. *n* = 3; ^#^ *p* < .05 versus control groups; *p < .05 versus STZ groups

Histopathological changes in the pancreatic tissues of the rats were examined to confirm the effect of *Gryllus bimaculatus* powder on pancreatic function and morphology. Degenerative and necrotic changes, as well as shrinkage of the islets of Langerhans, were observed in histological sections of pancreatic tissues from the STZ‐induced diabetes group (Figure 3a, top). However, *Gryllus bimaculatus* powder ameliorated this damage, especially at the highest dose (6.5 g/kg), and elicited a clear recovery in the STZ‐induced diabetes group. Furthermore, in immunohistochemical staining of the pancreatic tissues, a powder dose‐dependent decrease in insulin expression was clearly observable (Figure 3a, bottom). The insulin staining intensity was quantified (Figure 3b) and suggested that *Gryllus bimaculatus* powder preserved pancreatic β‐cell function and maintained pancreatic structure.

This study focused on the protective effects of *Gryllus bimaculatus* powder against changes in pancreatic morphology, that is, pancreatic damage. Administration of the *Gryllus bimaculatus* powder rescued both the reduced insulin staining and the changes in pancreatic structure (Figure 3a,b). STZ‐induced diabetic states result in excessive oxidative stress and pancreatic damage (Olatunji, Chen, & Zhou, 2017; Punithavathi, Prince, Kumar, & Selvakumari, 2011; Sakuraba et al., 2002). Previous reports have shown significant inhibition of mTORC1, accompanied by a reduction in β‐cell mass and decreased glucose tolerance, in an STZ‐injected model with rapamycin (Feng et al., 2017; Yoo & Park, 2018). Moreover, the mTOR‐regulated transcriptional network was also shown to play a key role in improving β‐cell survival and glucose homeostasis in diabetics (Chau et al., 2017; Feng et al., 2017; Wu et al., 2011; Yoo & Park, 2018). In this study, an mTOR‐related mechanism against diabetic dysfunction was applied to the nutrient‐based *Gryllus bimaculatus* interpretation (Table S1). Generally, nutrient‐rich conditions facilitate protein and lipid synthesis, as well as assimilation of mitochondrial metabolism, to drive intracellular and extracellular signals and assembly of the mTORC1 complex to regulate cell size, growth, and proliferation (Ali, Devkota, Roh, Lee, & Lee, 2016; Avila‐Flores, Santos, Rincon, & Merida, 2005; Duran et al., 2011; Laplante & Sabatini, 2009, 2012; Zoncu, Efeyan, & Sabatini, 2011).

### 3.4 Effects of *Gryllus bimaculatus* powder on pancreas damage resulting from STZ‐induced diabetes

**Figure 4:**
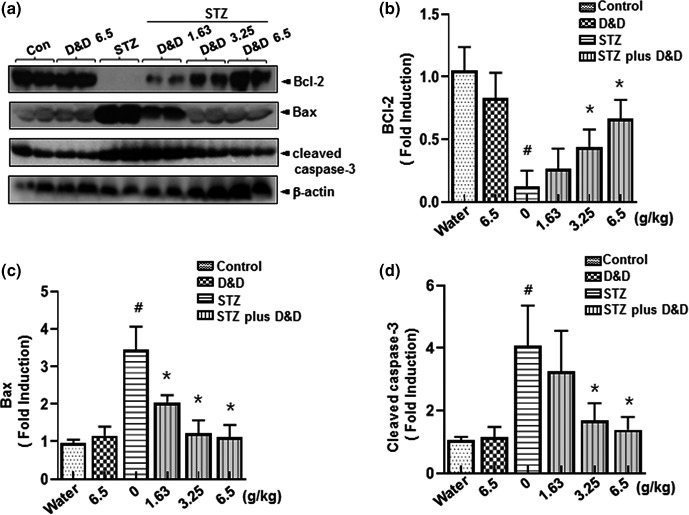
Effects of *Gryllus bimaculatus* powder on pancreas damage resulting from STZ‐induced diabetes. Eight‐week‐old rats were injected intraperitoneally with a single dose of freshly prepared STZ (65 mg/kg). The control group was injected with an equal volume of citrate buffer only. After confirming the diabetic condition, the control group was fed a high dose of *Gryllus bimaculatus* powder (6.5 g/kg), whereas the diabetic group was fed varying doses of the powder (1.63, 3.25, and 6.5 g/kg) twice daily. Immunoblotting with anti‐Bcl2, anti‐Bax, and anti‐cleaved caspase 3 antibodies was performed on pancreases isolated from the vehicle‐administered and *Gryllus bimaculatus* powder‐administered nondiabetic rats and from the vehicle‐administered and *Gryllus bimaculatus* powder‐administered diabetic rats, as described in the Materials and Methods (a). Quantification analysis of Bcl2 (b), Bax (c), and cleaved caspase 3 expression (d). Results were standardized to the expression of β‐actin at the indicated administered weight. Values are means ± *SD*. *n* = 3; ^#^ *p* < .05 versus control groups; *p < .05 versus STZ groups

We examined how *Gryllus bimaculatus* powder regulates β‐cell replication and survival in STZ‐induced diabetes. To investigate the effect of *Gryllus bimaculatus* powder on the apoptosis pathway in the pancreas, immunoblotting was performed to confirm the expression of the proapoptotic Bax and antiapoptotic Bcl2 proteins (Yoo & Park, 2018; Zhang et al., 2014). *Gryllus bimaculatus* powder treatment resulted in increased Bcl2 expression, decreased BAX expression, and inhibition of caspase 3 cleavage in the STZ‐induced diabetes group (Figure 4a). Quantification analysis showed the antiapoptotic role of *Gryllus bimaculatus* powder in pancreases from the STZ‐induced diabetes groups (Figure 4b–d). These findings suggest that the effects of the *Gryllus bimaculatus* powder against the pancreatic function and morphological alterations are well correlated with the anti‐ and proapoptotic protein expression pattern.

### 3.5 Effects of *Gryllus bimaculatus* powder on mTOR signaling in an STZ‐induced diabetes model

**Figure 5:**
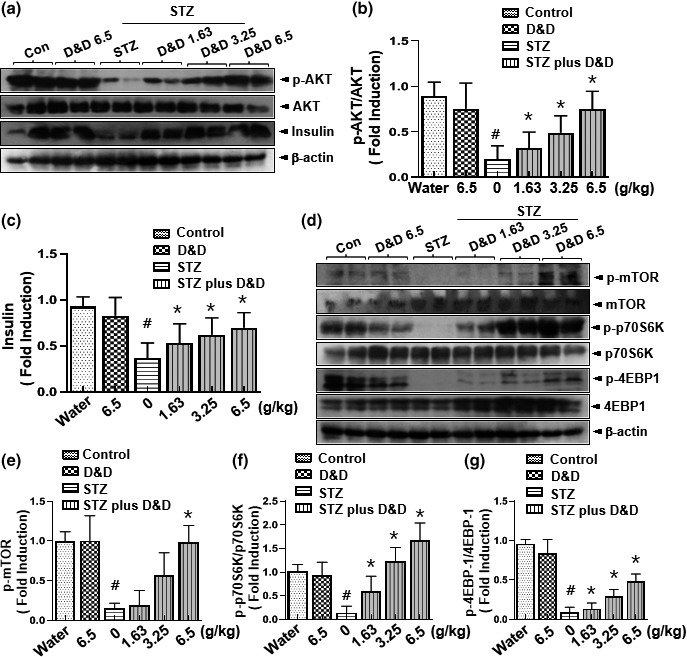
Effects of *Gryllus bimaculatus* powder on mTOR signaling in STZ‐induced diabetic rats. Eight‐week‐old rats were injected intraperitoneally with a single dose of freshly prepared STZ (65 mg/kg). The control group was injected with an equal volume of citrate buffer only. After confirming the diabetic condition, the control group was fed with a high dose of *Gryllus bimaculatus* powder (6.5 g/kg), whereas the diabetic group was fed with varying doses of the powder (1.63, 3.25, and 6.5 g/kg) twice daily. Immunoblotting with anti‐p‐AKT, anti‐AKT, anti‐insulin, and anti‐β‐actin antibodies was performed in pancreases isolated from the vehicle‐administered and *Gryllus bimaculatus* powder‐administered nondiabetic rats and from the vehicle‐administered and *Gryllus bimaculatus* powder‐administered diabetic rats (a). Quantification of p‐AKT (b) and insulin (c) expression based on the separate expression of AKT and β‐actin at the indicated time point. Immunoblotting with anti‐p‐mTOR, anti‐mTOR, anti‐p‐p70S6K, anti‐p70S6K, anti‐p‐4EBP1, anti‐4EBP1, and anti‐β‐actin antibody was performed with the same samples (d). Quantification of the expression of mTORC1 downstream signaling (e), p‐p70S6K (f), and p‐4EBP1 (g) based on the separate expression of p70S6K and 4EBP1. Values are means ± *SD*. *n* = 3; ^#^ *p* < .05 versus control groups; *p < .05 versus STZ groups

Because AKT status and mTORC1 activity are correlated with β‐cell mass and glucose tolerance (Chau et al., 2017; Feng et al., 2017; Yoo & Park, 2018), AKT signaling in the pancreas was examined in STZ‐induced diabetic rats treated or not with the *Gryllus bimaculatus* powder. As expected, STZ abrogated AKT activity and insulin, whereas the *Gryllus bimaculatus* powder rescued AKT activity and insulin to almost the control levels (Figure 5a–c). mTORC1 activity, associated with AKT cellular signaling, was also evaluated. The reduced mTORC1 activity, evidenced by the decreased patterns of p‐mTOR, p‐p70S6K, and p‐4EBP1 in the STZ‐induced diabetes group, was significantly rescued in the powder‐treated groups (Figure 5d–g).

Administration of extracts containing these compounds was shown to lower blood glucose levels and exert a regenerating effect on β‐cell loss. Compared to the reported beneficial effects, however, less is known of the mechanisms underlying the effects of the extracts. The results of this study suggested that AKT/mTOR signaling mediates STZ‐induced T1D. Evidence for the involvement of the AKT‐mTORC1 axis in controlling pancreatic homeostasis in this study includes the almost complete rescue of p‐mTOR, p‐p70S6K, and p‐4EBP1 with *Gryllus bimaculatus* powder treatment (Figure 5), indicating that *Gryllus bimaculatus* powder may be a promising material to enhance AKT and mTORC1 signaling in the diabetic condition.

## 4 CONCLUSIONS

In this study, *Gryllus bimaculatus* powder was suggested to have some beneficial effects against T1D, as evidenced by the results from a streptozotocin (STZ)‐induced diabetes rat model. Administration of this insect extract significantly rescued the reduced C‐peptide and insulin responses found in diabetic conditions. Moreover, the antiapoptotic protein Bcl2 and the proapoptotic proteins Bax and cleaved caspase 3 recovered to a control level in a dose‐dependent manner. The *Gryllus bimaculatus* powder regulated mTORC1, a master controller of nutrient sensing and cell growth, which we propose as a mechanism involved in the *Gryllus bimaculatus* powder‐induced glucose‐lowering effect in this model. In this study, *Gryllus bimaculatus* powder is suggested to be an effective natural functional food that exhibits the glucose‐lowering effect in STZ‐induced diabetic condition.

## CONFLICT OF INTEREST

The authors declare that they do not have any conflict of interest.

## ETHICAL APPROVAL

All animal procedures were conducted in accordance with the Principles of Laboratory Animal Care of the Association for Assessment and Accreditation of Laboratory Animal Care International (AAALAC) of Chonbuk National University Hospital (approval no: cuh‐IACUC‐2017–21) and were accredited by AAALAC on 8 November 2017 (cuh‐IACUC‐170316–6).
