# Supplementary material for: Glucose‐lowering effect of Gryllus bimaculatus powder on streptozotocin‐induced diabetes through the AKT/mTOR pathway
Source: Food Sci Nutr. 2019 Dec 11;8(1):402–9. doi: 10.1002/fsn3.1323 (PMC6977414; doi:10.1002/fsn3.1323)
Supplement: Supplementary file 1 [file FSN3-8-402-s001.docx]

Supplementary Table

Table 1. Analysis result of *Gryllus bimaculatus* powder (D&D_1)

| Contents | Result |
| --- | --- |
| Amino acid (Tyrosine)(mg/100g) | 2371.21mg/100g |
| Amino acid (Glycine)(mg/100g) | 2840.71mg/100g |
| Amino acid (Serine)(mg/100g) | 2419.57mg/100g |
| Amino acid (Alanine)(mg/100g) | 4845.04mg/100g |
| Amino acid (Glutamic Acid)(mg/100g) | 6802.79mg/100g |
| Amino acid (Lysine)(mg/100g) | 1228.55mg/100g |
| Amino acid (Leucine)(mg/100g) | 3650.78mg/100g |
| Amino acid (Methionine)(mg/100g) | 726.60mg/100g |
| Amino acid (Valine)(mg/100g) | 2694.40mg/100g |
| Amino acid (Arginine)(mg/100g) | 3265.57mg/100g |
| Amino acid (Asparatic acid)(mg/100g) | 4243.69mg/100g |
| Amino acid (Isoleucine)(mg/100g) | 1935.28mg/100g |
| Amino acid (Threonine)(mg/100g) | 1912.02mg/100g |
| Amino acid (Phenylalanine)(mg/100g) | 1560.92mg/100g |
| Amino acid (Proline)(mg/100g) | 2983.58mg/100g |
| Amino acid (Histidine)(mg/100g) | 1477.02mg/100g |
| Amino acid (Cystine)(mg/100g) | 149.85mg/100g |
| Amino acid (Tryptophan)(mg/100g) | 356.96mg/100g |
| Lauric acid (g/100g) | 0.03g/100g |
| Myristic acid (g/100g) | 0.27g/100g |
| Palmitic acid (g/100g) | 9.35g/100g |
| Stearic acid (g/100g) | 2.38g/100g |
| cis-5,8,11,14,17-Eicosapentaenoic acid (g/100g) | 0.10g/100g |
| cis-4,7,10,13,16,19-Docosahexaenoic acid (g/100g) | Non detected |
| Linolenic acid (g/100g) | Non detected |
| Linoleic acid (g/100g) | 14.55g/100g |
| Conjugated linoleic acid (mg/100g) | 42.98mg/100g |
| Arachidonic acid (g/100g) | 0.04g/100g |
| Oleic acid (g/100g) | 11.75g/100g |
| Potassium (mg/100g) | 1458.96mg/100g |
| Calcium (mg/100g) | 118.37mg/100g |
| Magnesium (mg/100g) | 113.78mg/100g |
| Zinc (mg/100g) | 14.80mg/100g |
| Selenium (μg/100g) | 39.74μg/100g |
| Iron (mg/100g) | 5.87mg/100g |
| Chromium (mg/100g) | 17.55mg/100g |
| Manganese (mg/100g) | 3.64mg/100g |
| Sodium (mg/100g) | 331.66mg/100g |
| Copper (mg/100g) | 1.44mg/100g |
| Vitamin A (μg RE/100g) | Non detected |
| Vitamin B1 (mg/100g) | 0.13mg/100g |
| Vitamin B2 (mg/100g) | 1.61mg/100g |
| Vitamin B6 (mg/100g) | 0.06mg/100g |
| Vitamin B12 (μg/100g) | 1.10μg/100g |
| Vitamin C (mg/100g) | Non detected |
| Vitamin D (μg/100g) | 1186.51μg |
| Vitamin E (mg α-TE/100g) | 1.90mg α-TE/100g |
| Niacin (mgNE/100g) | 15.23mgNE/100g |
| Biotin (μg/100g) | 612.96μg/100g |
| Folic acid (μg/100g) | 330.59μg/100g |
| Calorie (Kacl/100g) | 466.87Kacl/100g |
| Carbohydrate (%) | 7.03% |
| Crude protein (%) | 58.95% |
| Crude fat (%) | 22.55% |
| Mositure (%) | 6.34% |
| Ash (%) | 5.13% |
| Total polyphenols (mg/100g) | 1136.49mg/100g |
| Cadmium (mg/kg) | 0.0542mg/kg |
| Arsenic (mg/kg) | 1.7814mg/kg |
| Lead (mg/kg) | 0.0764mg/kg |
| Mercury (mg/kg) | 0.024mg/kg |
